# Forensic considerations in cases of fatal constipation

**DOI:** 10.1007/s12024-025-00950-8

**Published:** 2025-02-12

**Authors:** Roger W Byard, Neil E I Langlois, Marianne Tiemensma

**Affiliations:** 1 Forensic Science, Adelaide, SA Australia; 2 Adelaide School of Biomedicine, The University of Adelaide, Level 2, Room N237, Helen Mayo North, Frome Road, Adelaide, SA 5005 Australia; 3 College of Medicine and Public Health, Flinders University, Adelaide, SA Australia

**Keywords:** Constipation, Megacolon, Bowel obstruction, stercoral colitis/ulceration, Peritonitis, Abdominal compartment syndrome, Venous thrombosis

## Abstract

Constipation is characterized by persistent difficulty in defecating. It is a common disorder in the community particularly affecting the elderly and those with intellectual disabilities and neuropsychiatric disorders. It is also caused by numerous medications including analgesic, antidepressant, antihypertensive and anticholinergic agents. It may be asymptomatic or it may produce abdominal pain/cramps, bloating, nausea and anorexia progressing to urinary incontinence and fecal impaction, or paradoxical diarrhea due to overflow. A wide range of mechanisms associated with constipation may result in death including bowel obstruction, stercoral colitis with ulceration, perforation and peritonitis, respiratory compromise, abdominal compartment syndrome and venous thrombosis with pulmonary thromboembolism. Constipation may exacerbate pre-existing diseases and treatments such as laxative and enemas may be lethal. The autopsy examination of a case with constipation and megacolon should take into account all of the pre-existing conditions, as well as the possibility of underlying disorders such as Hirschprung disease. Review of the decedent’s medical and drug history and level of supportive care will be important. Toxicological evaluations may be useful.

## Introduction

Although it is recognised that there is considerable variability in the timing and regularity of individual human fecal excretion, constipation has been defined clinically as a situation where less than three stools are passed per week, or there is persistent difficulty in defecating. The Rome criteria specify that there should be two or more of the following for at least 12 weeks in the preceding year:

- Less than 3 defecations per week.
- For more than 25% of defecations:
  - ○ Straining.
  - ○ Lumpy or hard stools.
  - ○ A sense of incomplete evacuation.
- For 25% of times a requirement for assisted defecation or manual evacuation [1].

Constipation can be primary (idiopathic or functional), secondary (related to comorbidities), or iatrogenic (associated with medications) (Table 1) [2]. While it is an extremely common condition in the community it may occasionally come to medicolegal attention when it has resulted in a fatal outcome. Such cases may also receive significant media attention if issues related to duty of care are raised [3]. Given the relative rarity of such events and the absence of descriptions in standard forensic texts, the following study and review was undertaken to explore the range of potential lethal mechanisms and to identify predisposing factors and conditions.

**Table 1:** Associations of lethal constipation

**Increased age**
**Neuropsychiatric conditions**
Dementia
Depression
Cerebrovascular disease
Parkinson disease
Multiple sclerosis
**Psychiatric illness**
Schizophrenia
Autism
Pica
**Intellectual disability**
**Cerebral palsy**
**Intestinal disease**
Anorectal stenosis
Neoplasia
Hirschprung disease
MEN IIb syndrome
Certain infections
**Drug use**
Analgesics
Antihypertensives
Antidepressants
Anticholinergics
Antacids
**Medical/metabolic/endocrine conditions**
Scleroderma
Hyperparathyroidism
Hypothyroidism
Amyloidosis
Diabetes mellitus
Chronic renal disease
**Elder/child abuse/neglect**
**Miscellaneous**
Immobility
Lack of access to toilets
Reduced dietary/fluid intake

## Epidemiology

Constipation is most commonly found in older individuals, women, black populations and in the lower socioeconomic classes [4–6]. Age has a strong correlation with the prevalence of constipation amongst the elderly (> 65 years) living in the community, occurring in 22% of that age group in New Zealand and 24% in Minnesota, United States (US) [1]. These numbers increase in residential care/nursing home facilities where laxative use is as high as 59–74% in residents compared to 20–30% of age-matched individuals in the community [1]. Part of the issue in elderly individuals in care may involve lack of regular toileting or appropriate facilities, lack of privacy and avoidance of humiliation in shared facilities, particularly if there is impaction with fecal incontinence [1, 7]. The constant seepage of mucus and feces makes management difficult and enhances the development of decubitus ulceration [7]. It has been estimated that there are approximately 2.5 million visits to physicians for constipation per year in the US [6]. Chronic constipation has been shown to be associated with an increased risk of poorer survival in the community [8].

Functional constipation is also a common and likely underestimated problem in the pediatric population. The median prevalence of constipation in children is estimated to be 12% [9], with the peak incidence of constipation occurring between 2 and 4 years of age when toilet training starts, with a common mechanism for developing functional constipation in young children being withholding behaviour [10].

## Etiology

The pathophysiology of constipation is complex and multifactorial and includes factors such as slow colonic transit time, genetic predisposition, lifestyle habits, and psychological distress, among others [11]. The association of older age with constipation is likely due to significant comorbidities and polypharmacy, in addition to poor diet and reduced fluid intake [4]. Almost a half of those with frailty syndrome (45%), which is characterized by reduced physical strength, slow ambulation and declining activity, exhaustion, significant unintentional weight loss and sarcopenia (decreased muscle mass) [12], have reported constipation as a health issue [1].

Constipation is also a major health issue in those with autism, cerebral palsy and learning disabilities [13]. Studies have shown that constipation was a health problem in almost 60% of individuals with multiple and profound disabilities, with 65% having been prescribed laxatives within the previous year [2, 14]. Although only 24.1% of those with Down syndrome have had constipation reported, a systematic review found that 90% had functional constipation [14, 15]. Under-reporting of symptoms is likely in those with intellectual disabilities and associated communication difficulties, and pain may be atypically manifested as a behavioural change with aggression and self-injury [16]. Predisposing factors again include reduced fluid intake, inadequate diets, insufficient exercise and medications [2]. Constipation is also associated with psychiatric illnesses such as schizophrenia [17].

A wide variety of medications may cause constipation involving both prescription and over-the-counter drugs. Specific drugs include analgesics (opiates, non-steroidal anti-inflammatory agents), antidepressants, antihypertensives (diuretics, calcium channel blockers) and anticholinergics (antidepressants, antipsychotics, antihistamines and antiparkinsonian drugs). Calcium and iron supplements and antacids may also be contributory [1, 4, 18–24].

Cerebrovascular disease, amyotrophic lateral sclerosis, Parkinson disease and multiple sclerosis may be complicated by lack of mobility, autonomic dysfunction and weakness leading to constipation. In addition, neuromuscular disorders may lead to atrophy of the diaphragm, and pelvic floor and abdominal wall musculature interfering with straining [7, 25]. Constipation may also be associated with the following conditions: scleroderma, hyperparathyroidism, hypothyroidism, amyloidosis, diabetes mellitus and chronic renal disease [1].

Other conditions such as neoplasia, spinal cord injuries or anorectal stenosis may impair fecal evacuation, and in children in particular the possibility of cystic fibrosis, Hirschprung disease or rare entities such as MENIIB (multiple endocrine neoplasia) syndrome must be considered [7, 26–30]. Infection with *Trypanosoma cruzi* results in Chagas disease which, in addition to effects on the nervous system and heart, may cause enteric nervous system impairment with approximately one third of patients developing dilation of the gastrointestinal tract including megacolon with constipation [31]. This has resulted in fecal impaction [7].

## Clinical manifestations

Constipation may be asymptomatic or it may produce abdominal pain/cramps, bloating, nausea and anorexia. There may be anal fissures, hemorrhoids and pelvic organ prolapse [16]. As it progresses there may be overflow incontinence resulting in diarrhea, urinary incontinence and fecal impaction [2]. There may be an association of megacolon with constipation-predominant irritable bowel syndrome [32, 33]. Further progression can lead to intestinal obstruction or perforation, and in some cases death [34].

Fecal impaction is defined as a large mass of compacted feces at any level in the intestine that cannot be evacuated spontaneously. It causes intraluminal pressure of the colon with resultant ischemic effects that can lead to ulceration and perforation. Other complications include megacolon, mechanical colonic obstruction, and mass effect on adjacent structures including nerves, blood vessels or solid organs [35].

## Forensic issues

A number of different mechanisms may be responsible for death from constipation which are summarized in Table 2.

**Table 2:** Potentially lethal mechanisms associated with constipation

**Bowel obstruction**
Mechanical obstruction
Volvulus
Feculent vomiting with aspiration
**Stercoral colitis**
Ulceration
Perforation with peritonitis
Sepsis
**Respiratory compromise**
**Abdominal compartment syndrome**
Multiorgan failure/sepsis
Leg ischemia
**Venous thrombosis with**
Pulmonary thromboembolism
**Exacerbation of underlying disease**
Synergistic effects
Enhanced atherogenesis
**Treatment associated**
Laxative use
Enemas
*Electrolyte disturbances*
*Intestinal perforation*

### Bowel obstruction

Filling of the colon with impacted feces can result in simple mechanical bowel obstruction which if untreated can lead to mucosal ulceration and sepsis, significant fluid and electrolyte sequestration, hypovolemic shock and lethal multiorgan failure. Elderly individuals are at particular risk and may not be diagnosed during life as they may not have manifested symptoms and signs [36]. In addition, problems in communication may exist because of underlying dementia or cerebrovascular disease, and the concomitant use of analgesic agents prescribed for other conditions such as arthritis may mask symptoms [36]. Those living a reclusive life style, so-called Diogenes syndrome, may have avoided seeking medical attention [37]. Mortality rates from obstruction in the elderly are as high as 16% [7].

**Fig. 1:**
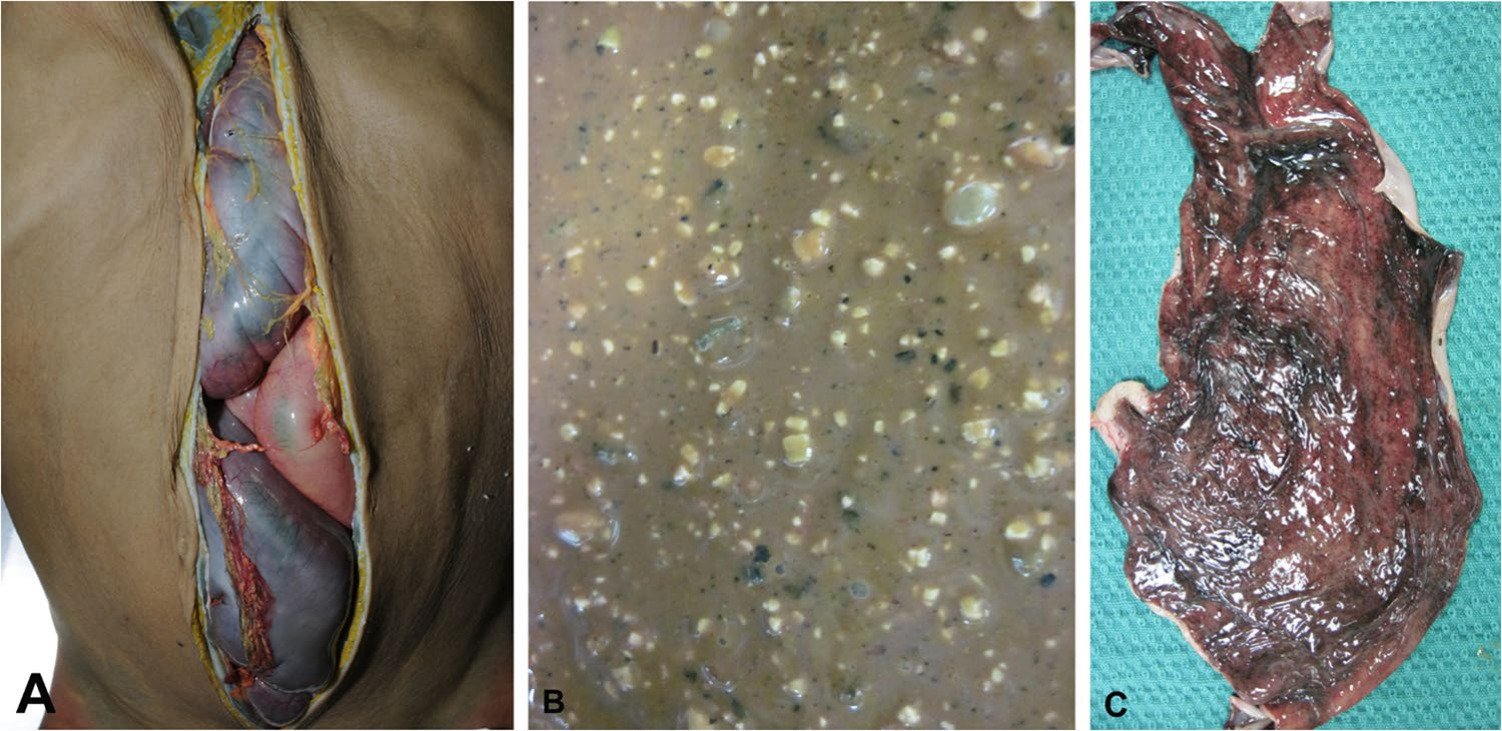
Opening of the abdominal cavity of a 9-year-old boy with pica showing bulging of a discoloured sigmoid volvulus (**A**). A sample of non-solid feces containing a mixture of uncooked rice, dirt and stones (**B**). Opening of the sigmoid colon showing mucosal ulceration and necrosis (**C**)

Constipation may also result in volvulus with twisting of segments of the colon which may acutely compromise intestinal blood flow causing ischemic necrosis, gangrene and sepsis, with or without perforation [36, 38]. Constipation is the leading cause of sigmoid volvulus in the elderly [38, 39], although it also occurs in children. For example, a 9-year-old boy with Goldenhar syndrome and pica has been reported who died from a sigmoid volvulus with ischemic intestinal necrosis due to filling of the colon with feces containing dirt, stones and rice (Fig. 1) [40]. Sigmoid volvulus may also be associated with Hirschprung disease [41]. All of these obstructive conditions may result in megacolon resulting in feculent vomiting with aspiration which may also be lethal.

### Stercoral colitis

**Fig. 2:**
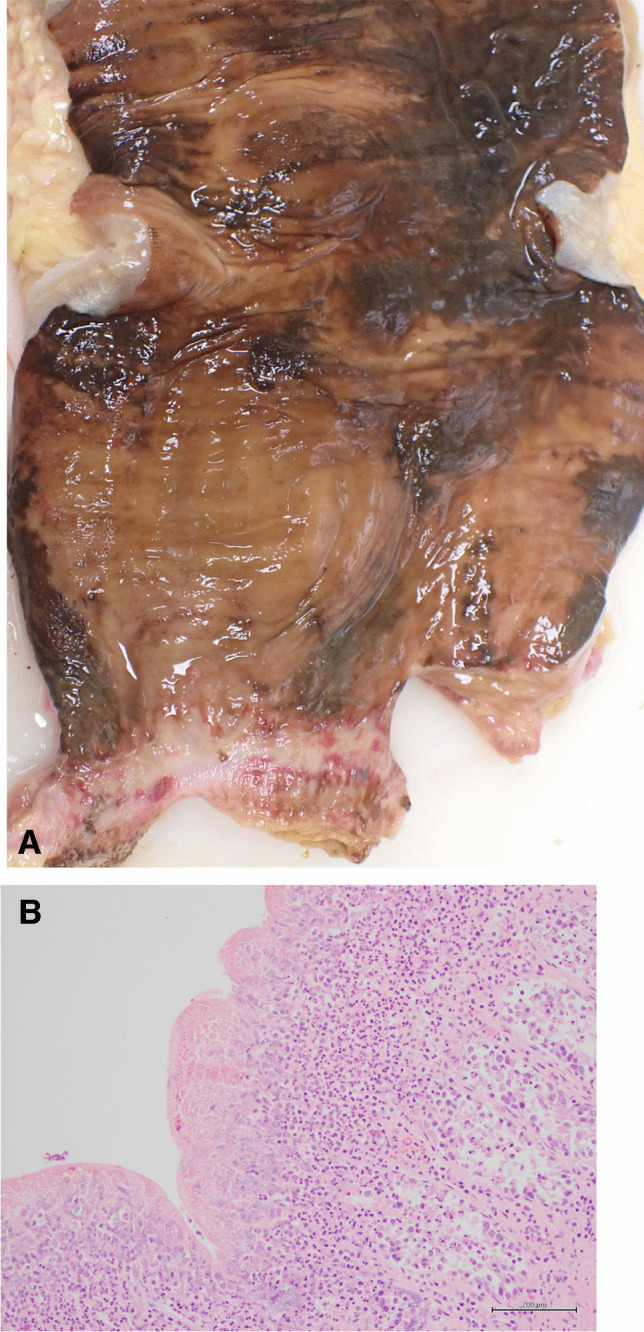
Opened rectum in an 11-year-old boy with a history of attention deficit hyperactivity disorder and autism who died from sepsis due to stercoral colitis/ulceration secondary to constipation. A hard 6 × 3 × 3 cm fecal mass had been removed from the rectum to show focal areas of stercoral ulceration (**A**). Histologic section of the colon from a 5-year-old boy with fecal impaction and multiple fecoliths showing florid stercoral colitis (Hematoxylin and Eosin x 200)

**Fig. 3:**
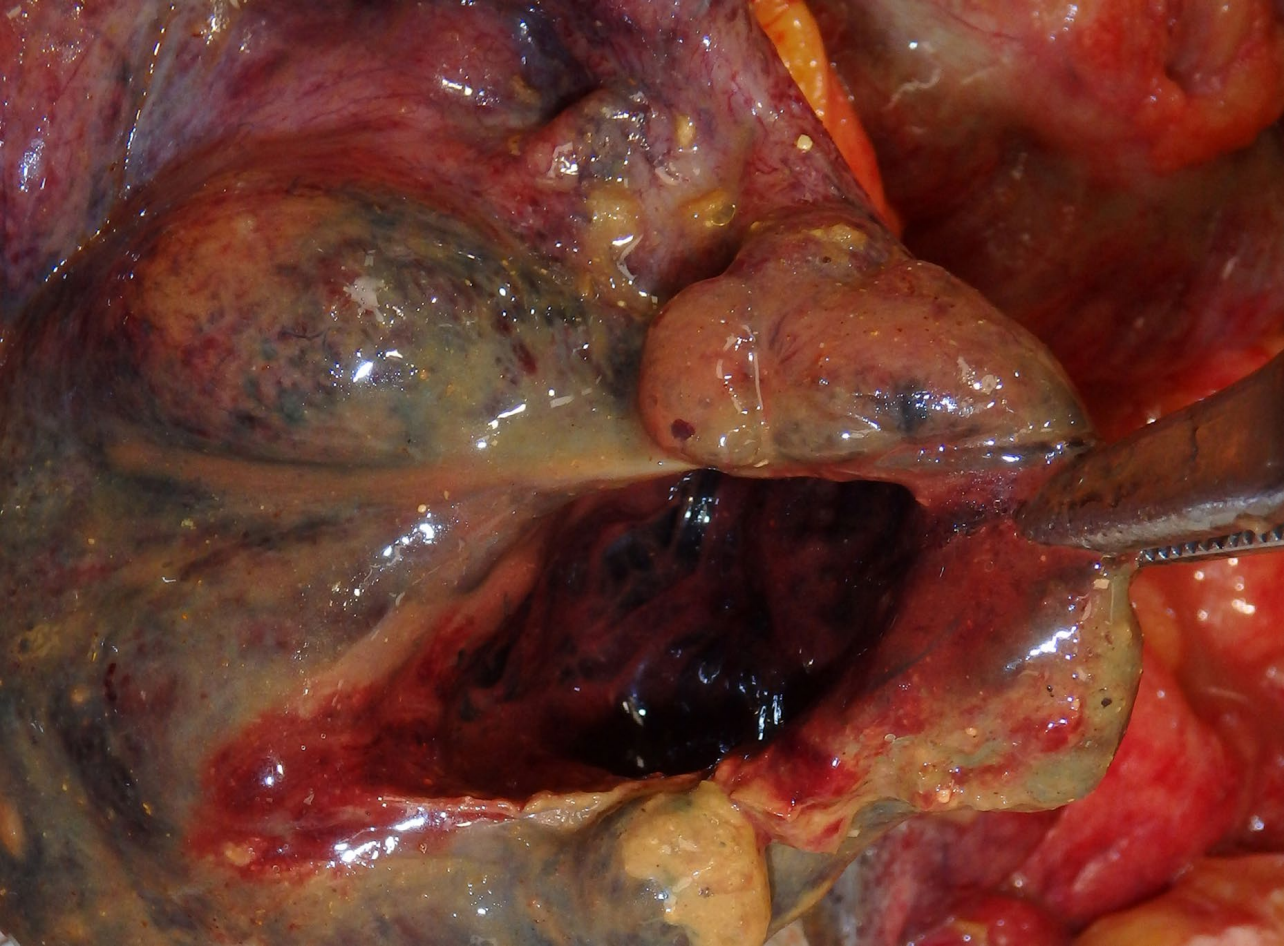
Stercoral perforation of the descending colon in a 64-year-old woman with a history of chronic back pain and migraines. The colon was filled with bulky firm fecal masses with a 40 × 40 mm perforation. The adjacent intestine was necrotic with a hemorrhagic peritonitis

Impacted feces may cause pressure necrosis of the colonic wall leading to inflammation with ischemic ulceration and possible perforation with peritonitis (Figs. 2 and 3) [16]. This usually affects the sigmoid colon and rectum [42]. Massive hemorrhage is rare although there may be occult bleeding. Delays in diagnosis are responsible for a high mortality rate from perforation in the elderly [7].

### Respiratory compromise

**Fig. 4:**
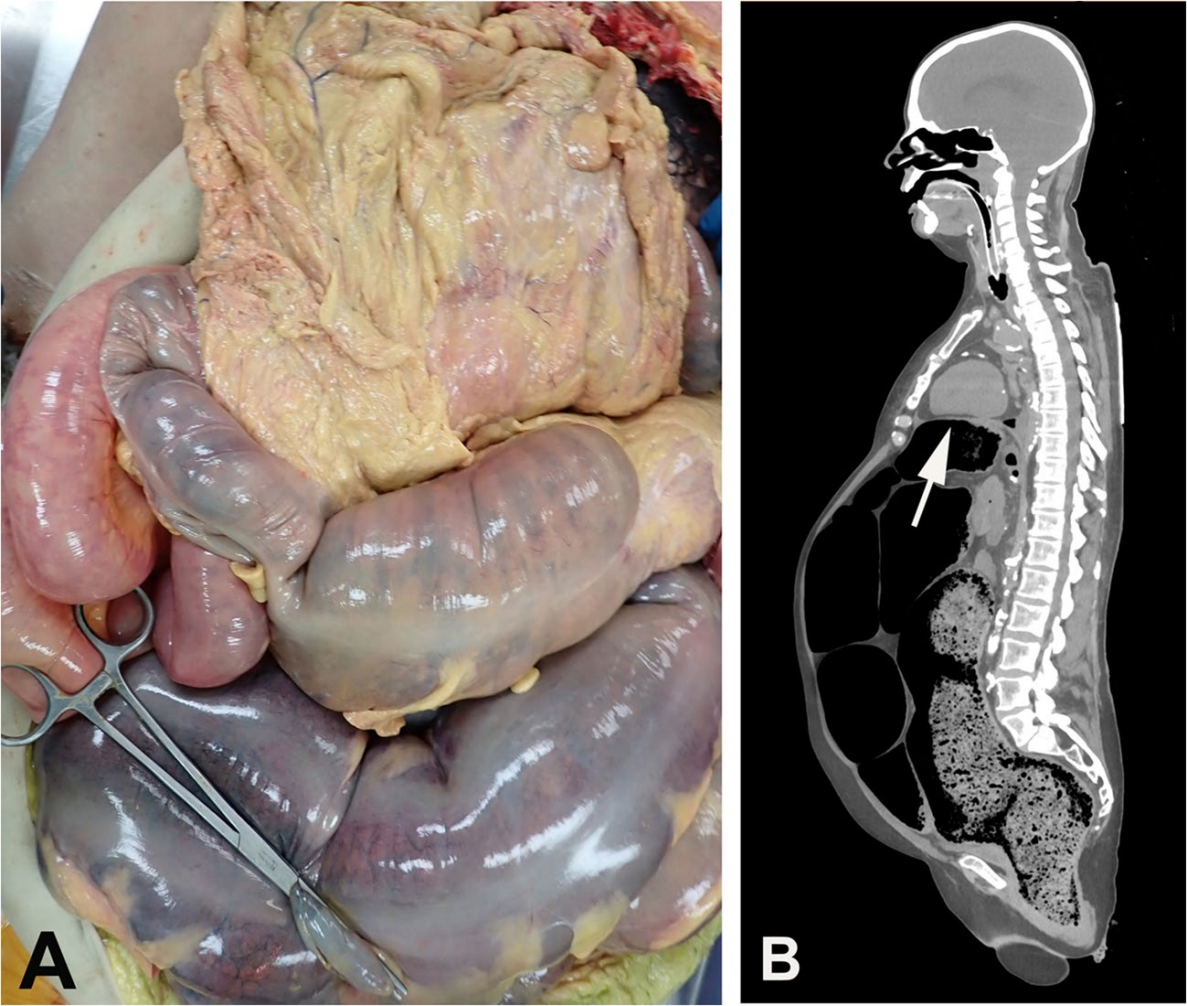
Markedly dilated colon containing 7.6 kg of soft feces in a 66-year-old woman with a history of bipolar disorder and constipation. [The clamp is closing a defect in the colonic wall caused when the abdominal cavity was opened] (**A**). PMCT showed significant elevation of the diaphragm with pleural space and lung compression (arrow) (**B**)

**Fig. 5:**
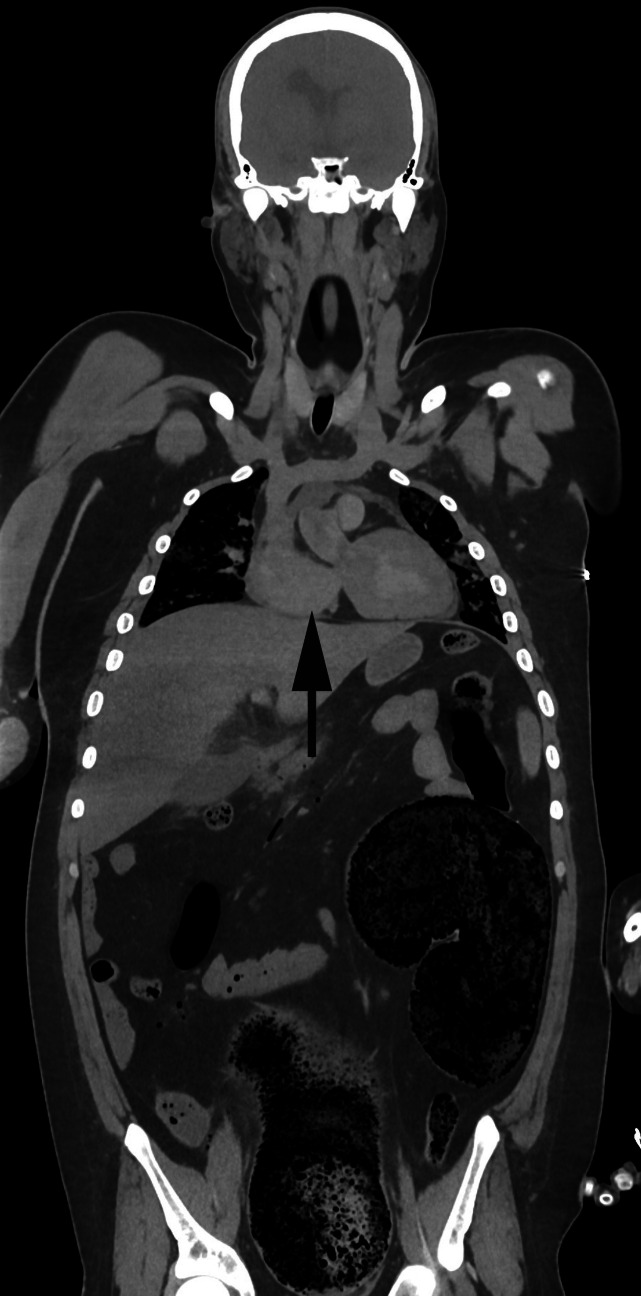
Elevation of the diaphragm with compression of chest organs (arrow) due to massive constipation in a 14 -year-old boy with a history of cerebral palsy and autism

Filling of the colon with feces resulting in megacolon will occupy considerable space within the abdominal cavity with upwards displacement of the diaphragm and compression of the pleural cavities and lungs (Figs. 4 and 5). This may also be complicated by aspiration of feculent material [43].

### Abdominal compartment syndrome

**Fig. 6:**
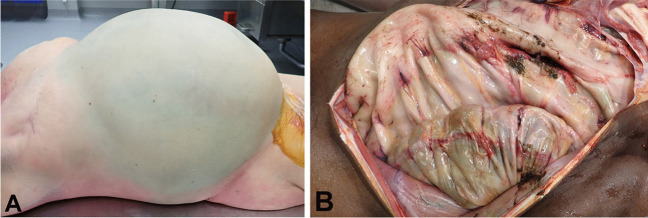
Markedly distended abdomen in a 66-year-old woman with impaction of 7.6 kg of feces (**A**). Opened abdominal cavity in a 17-year-old male who died of abdominal compartment syndrome due to chronic constipation with acquired megacolon and megarectum (**B**)

Abdominal compartment syndrome results from a significant increase in pressure within the abdominal cavity compressing the vena cava with subsequent metabolic abnormalities and cardiogenic shock [44, 45]. The renal, respiratory, gastrointestinal and hepatic systems may also be affected [46]. While there are a number of causes of the syndrome it may result from megacolon due to chronic constipation. Such was the case of a 17-year-old male with intellectual disability and chronic constipation who died of abdominal compartment syndrome and who was found at autopsy to have a very tense distended abdomen with a megacolon and megarectum containing 6.5 kg of dark brown semi-solid feces (Fig. 6) [13].

### Venous thrombosis with pulmonary thromboembolism

**Fig. 7:**
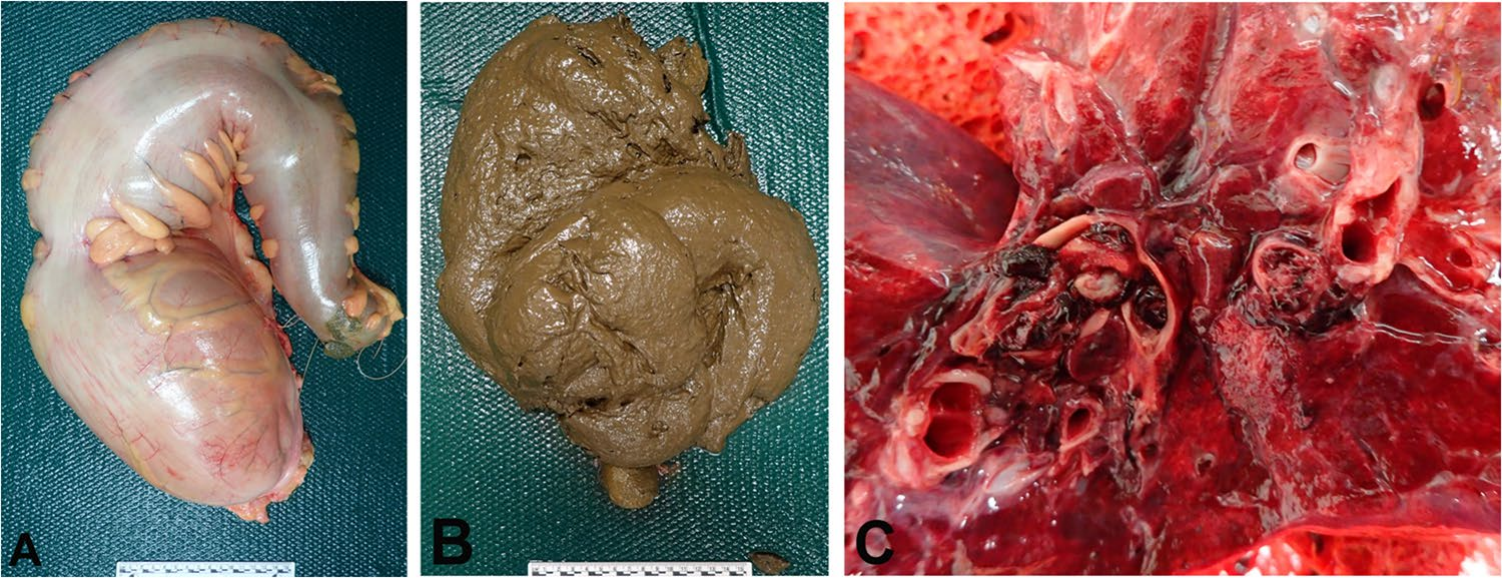
Hugely dilated rectum and sigmoid colon (**A**) containing 4.6 kg of soft brown feces (**B**) removed from a 17-year-old male who died of pulmonary thromboembolism (**C**) arising from compression of pelvic veins by acquired megacolon due to constipation

It has been shown in a population-based study of 83,239 patients with constipation in Denmark that there was a significantly increased incidence of venous thromboembolism. This was in part related by the authors to risk factors of obesity and inactivity [47, 48]. Another possibility is simple mechanical compression of pelvic veins by a markedly enlarged colon [49] as it has been shown to occur with other space occupying masses such as uterine and ovarian tumors, endometriosis, pregnancy, bladder distention (from benign prostatic hypertrophy or a neurogenic bladder), synovial cysts, psoas abscesses and penile prosthetic reservoirs [50]. This was the cause of death in a 17-year-old male with a past history of cerebral palsy and autism who was found unexpectedly dead. At autopsy pulmonary thromboembolism was found attributed to compression of pelvic veins by acquired megacolon due to constipation (Fig. 7) [13].

### Exacerbation of underlying disease

As was noted in the above Danish study, constipated individuals had a greater risk of venous thrombosis than controls. In addition, there were higher rates of myocardial infarction, ischemic and hemorrhagic strokes and peripheral vascular disease [47, 51]. Constipation has been associated with high blood pressure variability [52]. Although the etiology was considered to be multifactorial the possibility of alterations in the gut microbiome from constipation was suggested as this has been associated with hypertension and increased atherogenesis [53, 54]. Constipated patients with heart failure also have a higher rate of hospital readmission [55].

### Treatment-associated

Fatal hypermagnesemia has been reported in constipated individuals from the consumption of magnesium-containing laxatives, Epsom salts ingestion and enemas [56]. Individuals with impaired renal function are at particular risk [57].

Enema administration may be associated with a range of potentially lethal events including rectal necrosis [58], rectal perforation [59] and fluid and electrolyte disturbances [60]. Children have been found to be at higher risk of adverse outcomes from hyperphosphate enema solutions [61] and the danger of hypotonic enemas in children with Hirschprung disease has been recognized for decades [62].

## Conclusions

This review has detailed the diverse etiology of chronic constipation and the wide range of lethal mechanisms that may be induced by fecal overload. At autopsy careful review of the medical history should be undertaken for neuropsychiatric and developmental disorders that may cause constipation, including the list of the decedent’s medications.

Examination should be undertaken for associated neurological, systemic and gastrointestinal conditions and the quality of supportive care should be assessed to identify cases of elder and child neglect. Although constipation is widespread in the community it may be associated with significant morbidity and mortality.

## Data Availability

There is no original data other than the deidentified images.
